# Gait Characteristics in Children with Juvenile Idiopathic Arthritis and Secondary Scoliosis

**DOI:** 10.3390/children13010083

**Published:** 2026-01-05

**Authors:** Gökçe Leblebici, Eylül Pınar Kısa, Ela Tarakcı, Özgür Kasapçopur

**Affiliations:** 1 Division of Physiotherapy and Rehabilitation, Faculty of Health Science, Istanbul Medeniyet University, Atalar, Şht. Hakan Kurban Cd. No:44, 34862 Istanbul, Turkey; 2 Department of Occupational Therapy, Istanbul Medipol University, 34810 Istanbul, Turkey; 3 Division of Physiotherapy and Rehabilitation, Faculty of Health Science, Istanbul University-Cerrahpasa, 34320 Istanbul, Turkey; 4 Department of Pediatric Rheumatology, Cerrahpaşa Faculty of Medicine, Istanbul University-Cerrahpasa, 34320 Istanbul, Turkey

**Keywords:** juvenile idiopathic arthritis, scoliosis, gait, postural asymmetry

## Abstract

**Highlights:**

**What are the main findings?**

- Children with juvenile idiopathic arthritis and scoliosis showed reduced forefoot loading and lower maximum plantar loading compared with age-matched healthy peers.
- These children also demonstrated shorter single-limb support on the dominant side and increased medio-lateral center of mass displacement, indicating altered trunk control during gait.

**What are the implications of the main findings?**

- Baropodometric gait analysis may help clinicians detect subtle gait and balance alterations in children with JIA-associated scoliosis that are not evident in routine clinical examination.
- The identified deviations in plantar loading and trunk stability can be used as hypothesis-generating information for designing and testing future targeted rehabilitation strategies in this population.

**Abstract:**

Background: Juvenile Idiopathic Arthritis (JIA) is a chronic inflammatory condition that can disrupt joint function and biomechanics, often leading to altered gait patterns. When coexisting with secondary scoliosis—a common musculoskeletal complication in children with JIA—postural and movement impairments may be further exacerbated. However, limited research has investigated the combined impact of JIA and secondary scoliosis on gait characteristics. This study aimed to evaluate gait parameters in children diagnosed with JIA and secondary scoliosis and to compare them with age-matched healthy peers. Methods: A total of 50 children (25 with JIA and secondary scoliosis, 25 healthy controls) were included. Demographic data, plantar pressure distribution, temporal gait parameters, and center of mass (CoM) displacement were assessed using computerized gait analysis. Group comparisons were performed using appropriate statistical methods. Results: Children with JIA and secondary scoliosis exhibited significantly lower forefoot loading on both dominant and non-dominant sides compared to controls (*p* < 0.05). Maximum loading values were also reduced bilaterally in the JIA group (*p* < 0.001). The dominant side single-limb support duration was significantly shorter (*p* = 0.027), and CoM displacement was greater (*p* = 0.044) in the JIA group. No differences were observed in rearfoot loading or walking speed. Conclusions: Children with coexisting JIA and secondary scoliosis demonstrate altered gait mechanics, likely reflecting compensatory adaptations due to joint inflammation and postural asymmetries. Gait analysis may offer valuable insights for tailoring rehabilitation strategies in this patient population.

## 1. Introduction

Juvenile Idiopathic Arthritis (JIA) is one of the most common chronic rheumatic diseases in childhood, affecting approximately 1 in 1000 children worldwide [1]. Characterized by persistent joint inflammation, JIA presents with symptoms such as swelling, stiffness, limited mobility, and pain [1,2]. These symptoms, especially chronic joint inflammation, can seriously affect daily activities and overall quality of life in children with JIA [3]. As the disease progresses, it often leads to changes in joint mechanics, placing stress not only on the involved joints but also on unaffected areas of the body. Even in unaffected joints, arthritis may change the loading, and the kinetic chain is modified by this [4].

One of the functional consequences of JIA is the disruption of normal biomechanical function [5]. Inflammation and structural changes in joints cause changes in movement patterns, particularly in the kinetic chain, the interconnected system of joints and muscles that work together to move the body [6]. This change in biomechanics can have far-reaching effects, including changes in posture and gait [7]. In some cases, these altered mechanics can lead to the development of musculoskeletal deformities such as secondary scoliosis, a lateral curvature of the spine that is frequently seen in children with JIA [8].

Scoliosis is characterized by three-dimensional deformity of the spine, which alters trunk alignment and rotational symmetry during static and dynamic tasks [9]. Gait studies in individuals with scoliosis have reported asymmetric trunk rotation relative to the pelvis, uneven weight distribution between limbs, and modifications in ground reaction forces, particularly in the mediolateral direction [10,11]. Such changes are thought to reflect compensatory strategies aimed at maintaining spinal stability and preventing further progression of the coronal and rotational deformity. Trunk imbalance and impaired postural control in scoliosis have also been associated with altered plantar pressure patterns and increased sway of the center of pressure during standing and walking [12].

Scoliosis can further complicate the clinical presentation of JIA, exacerbating problems with posture, balance, and mobility [4,13,14]. In addition, the presence of both JIA and secondary scoliosis raises important questions about how these conditions affect gait. Several studies using three-dimensional gait analysis and plantar pressure platforms have demonstrated altered joint kinematics, reduced walking speed and cadence, and abnormal plantar pressure distribution in this population [7,15,16]. Gait analysis in children with JIA often reveals deviations from normal movement patterns [7,17,18,19], but the specific factors contributing to these abnormalities, particularly in those with secondary scoliosis, are poorly understood. Inflammation-related changes in joint structures and periarticular soft tissues can lead to limited range of motion, muscle weakness, and altered proprioception, which together disturb the normal sequencing of the kinetic chain during walking [20]. In particular, ankle and midfoot involvement may promote pes planus, medial collapse, and shifts in plantar pressure towards more proximal regions of the foot, while knee and hip impairments contribute to increased hip flexion, internal rotation, and crouch-like gait patterns [21]. These biomechanical alterations are closely linked to changes in center of pressure progression and may secondarily affect trunk posture and dynamic balance. Although the literature acknowledges the potential for altered gait in children with JIA [15,17,18,19,22], the complex relationship between joint disease, secondary scoliosis, and gait mechanics has not yet been fully explored.

This study aimed to examine gait characteristics in children with JIA and secondary scoliosis and to investigate differences in walking compared to healthy individuals.

## 2. Materials and Methods

### 2.1. Study Design

This study is designed as a single-center, prospective and non-randomized controlled study investigating the walking behavior in children with JIA and secondary scoliosis and to investigate differences in walking compared to healthy individuals. The study was conducted in accordance with the Declaration of Helsinki and approved by the Local Ethics Committee (Istanbul Medipol University, Non-interventional Studies Ethics Committee/2024-1228). As the participants were under 18 years old, written informed consent was obtained from each patient’s parent.

### 2.2. Participants

The target population for this study consisted of children with oligoarticular JIA and secondary scoliosis, and age-matched healthy volunteers. The participants who applied to the Istanbul University-Cerrahpasa Faculty of Medicine Hospital between December 2024 and February 2025, had a diagnosis of oligoarticular JIA for at least six months based on the Pediatric Rheumatology International Trials Organization (PRINTO) criteria [23], and reported stable clinic symptoms in the past month were included in the study.

All children with JIA were under regular follow-up in the pediatric rheumatology clinic and had a confirmed oligoarticular JIA diagnosis for at least six months. According to the treating rheumatologist, all participants in the JIA group were clinically stable, without a recent flare or escalation of pharmacological treatment during the month preceding the gait assessment. Children who required an increase in anti-rheumatic medication because of active disease were not scheduled for gait analysis until a stable clinical status was achieved.

25 children (11.76 ± 2.26 years) with secondary scoliosis diagnosed with JIA and 25 healthy age-matched children were included in the study. A diagnosis of JIA, age between 8 and 16 years, a Cobb angle on a spine X-ray between 10° and <45°, and skeletal maturity rated between 0 and 4 on the Risser scale were all requirements for inclusion. A history of spinal surgery, lower extremity asymmetry, prior scoliosis treatment, cognitive impairments that would make it difficult to comprehend the questions posed, and any other neurological or orthopedic condition other than secondary scoliosis linked to JIA that might impact treatment results were all considered exclusion criteria.

This study was exploratory in nature, and the sample size was based on similar previous studies investigating gait in pediatric populations with JIA [7,18]. While no formal power analysis was conducted, the sample size is consistent with prior work in the field.

### 2.3. Outcome Measures

In addition to routine clinical examination, the pediatric rheumatologist documented the presence of active joints and overall clinical impression of disease stability at the time of testing. Although formal composite disease activity scores (such as the Juvenile Arthritis Disease Activity Score) and detailed pharmacological regimens were not systematically collected for research purposes in this study, only children judged to be in a clinically stable condition during the previous month were included in the JIA group.

The Cobb angle was assessed to evaluate the spinal curvature. The measurements were taken from a standing posteroanterior spine radiograph. Lines defining the scoliosis were drawn parallel to the superior border of the most deviated vertebra and the inferior border of the least deviated vertebra. Perpendicular lines to these were then drawn, and the angle formed at their intersection was recorded [24]. Radiological imaging and Cobb angle measurements were conducted by the physician. The Risser scale was used to grade skeletal maturity from 0 to 4 based on the degree of ossification of the iliac apophysis; lower Risser grades indicate greater remaining growth potential and a higher likelihood of scoliosis progression [24].

Angle of Trunk Rotation (ATR) was assessed to evaluate gibbosity using a Bunnell scoliometer. The measurement was performed by placing the scoliometer vertically along the axial axis of the spine, perpendicular to the spinous processes of the vertebrae. The assessment was conducted in a standing position with the participant bending forward, arms extended. The scoliometer was moved from the thoracic region to the sacrum, and the largest angle of rotation in the major curvature was recorded [25].

Center of mass (CoM) displacement (millimeter), single-limb stance duration (SLS) (millisecond), foot loading in forefoot and rearfoot (per cent), and walking speed (meter/second) during gait were obtained using the FreeMed baropodometry platform (the FreeStep v. 1.0. 3 88 software, Sensor Medica, Guidonia Montecelio, Roma, Italy). Participants were instructed to walk on a 10 m walkway at their preferred speed. Prior to the assessment, they were given a one-minute familiarization period to walk on the walkway. All plantar pressure and temporal parameters were normalized to body weight (for loading variables) and to gait cycle duration (for temporal variables) to allow for comparison between participants with different anthropometric characteristics.

For dynamic plantar pressure analysis, the software automatically segmented each footprint into forefoot and rearfoot regions based on standardized anatomical masks referenced to foot length. Forefoot loading and rearfoot loading were defined as the percentage of total plantar load (expressed as % of body weight) recorded under the forefoot and rearfoot regions, respectively, during the stance phase. Overall loading was calculated as the proportion of total plantar load borne by each limb (dominant and non-dominant), providing an index of weight-bearing symmetry between sides. Maximum loading represented the peak instantaneous load (as % of body weight) recorded under each foot during stance. SLS duration was derived from the temporal gait cycle as the time interval (milliseconds) during which only one foot was in contact with the platform, corresponding to the single support phase of gait for the dominant and non-dominant limbs. Walking speed (km/h) was calculated automatically by the system. The CoM displacement was calculated automatically by the FreeStep software based on the trajectory of the CoM during the stance phase and the distribution of plantar pressures under the feet. The software computes CoM excursion in the medio-lateral direction and provides the magnitude of displacement in millimeters over the dominant and non-dominant sides.

During the baropodometric assessment, each participant performed a series of walking trials along the 10 meter walkway, with the platform positioned in the middle of the path. To ensure reliable measurement, children completed a minimum of three and a maximum of five valid trials at their self-selected comfortable speed. A trial was considered valid if the child walked naturally without targeting the platform and maintained a consistent walking pattern throughout the walkway. During testing, participants were instructed to walk barefoot along the walkway, looking straight ahead, without talking, and at their usual comfortable pace. They were asked not to alter their step length or cadence to “hit” the platform, and a short familiarization period was provided before data collection to reduce adaptation effects. Children who appeared distracted or who interrupted their walking pattern were asked to repeat the trial.

### 2.4. Statistical Analysis

SPSS version 24 (Statistical Package for the Social Sciences) was used to perform statistical analyses. Descriptive statistics for numerical data were presented as mean ± standard deviation, while categorical data were expressed as frequency and percentage. The distribution of continuous variables was examined visually (histograms and Q–Q plots) and analytically using the Shapiro–Wilk test. For variables that met normality and homogeneity of variance assumptions, between-group comparisons were conducted using the independent samples Student’s *t*-test. When the distribution of a variable deviated from normality, non-parametric tests (Mann–Whitney U) were considered; however, inspection of the data indicated that key gait parameters were approximately normally distributed in both groups, and therefore the t-test was retained for the primary analyses. For key outcomes, 95% confidence intervals (CI) for between-group differences are reported in the Results. In addition, a brief post hoc power consideration was conducted for selected gait parameters using the observed effect sizes to evaluate whether the study was reasonably powered to detect between-group differences. Statistical significance was defined as *p* < 0.05 [26].

## 3. Results

A total of 50 participants were included in the study, comprising 25 children diagnosed with JIA and secondary scoliosis and 25 age-matched healthy controls. The two groups were similar in terms of sex distribution (JIA: 72% female, controls: 64% female; *p* = 0.54), age (JIA: 11.76 ± 2.26 years; controls: 11.42 ± 2.67 years; *p* = 0.57), and BMI (JIA: 20.18 ± 2.19 kg/m^2^; controls: 19.12 ± 3.11 kg/m^2^; *p* = 0.53) (Table 1).

Compared to healthy controls, children with JIA and secondary scoliosis exhibited significantly lower forefoot loading on both the dominant (15.95 ± 8.36% vs. 19.32 ± 8.77%, mean difference −3.37%, 95% CI −10.44 to 3.70; *p* = 0.028) and non-dominant sides (15.60 ± 8.76% vs. 19.88 ± 8.82%, mean difference −4.28%, 95% CI −11.54 to 2.98; *p* = 0.026). Rearfoot loading did not differ significantly between groups for either the dominant (33.96 ± 10.11% vs. 34.16 ± 7.82%; mean difference −0.20%, 95% CI −7.66 to 7.26; *p* = 0.611) or non-dominant side (34.47 ± 9.67% vs. 35.44 ± 8.65%; mean difference −0.97%, 95% CI −8.54 to 6.60; *p* = 0.242). Overall loading was similar across groups on both sides (dominant: 49.92 ± 6.36% vs. 51.54 ± 8.18%; mean difference −1.62%, 95% CI −7.67 to 4.43; *p* = 0.590; non-dominant: 50.02 ± 6.38% vs. 50.71 ± 6.21%; mean difference −0.69%, 95% CI −5.89 to 4.51; *p* = 0.133) (Table 2).

Children with JIA and secondary scoliosis had significantly lower maximum loading values on both the dominant (83.26 ± 20.60% vs. 85.47 ± 24.56%; mean difference −2.21%, 95% CI −20.92 to 16.50; *p* = 0.041) and non-dominant sides (80.12 ± 18.55% vs. 84.27 ± 23.15%; mean difference −4.15%, 95% CI −21.47 to 13.17; *p* = 0.042) than their healthy peers. In terms of temporal parameters, SLS duration was significantly shorter in the JIA group on the dominant side (355.16 ± 61.26 ms vs. 371.19 ± 60.88 ms; mean difference −16.03 ms, 95% CI −66.45 to 34.39; *p* = 0.027), while no significant difference was found on the non-dominant side (347.60 ± 59.19 ms vs. 354.59 ± 62.20 ms; mean difference −6.99 ms, 95% CI −57.12 to 43.14; *p* = 0.081). Walking speed did not differ significantly between groups (4.73 ± 0.88 km/h vs. 4.98 ± 1.25 km/h; mean difference −0.24 km/h, 95% CI −1.14 to 0.65; *p* = 0.862) (Table 2).

Dominant side CoM displacement was significantly greater in the JIA group (186.32 ± 24.98 mm) compared to the control group (171.31 ± 28.67 mm; mean difference 15.01 mm, 95% CI −7.19 to 37.21; *p* = 0.044). No significant difference was observed on the non-dominant side (174.22 ± 37.47 mm vs. 175.19 ± 35.97 mm; mean difference −0.97 mm, 95% CI −31.29 to 29.35; *p* = 0.841) (Table 2).

## 4. Discussion

This study aimed to investigate gait behavior in children diagnosed with JIA and secondary scoliosis, focusing on plantar pressure distribution, loading characteristics, temporal gait parameters, and CoM displacement. The results revealed significant differences in gait mechanics between children with JIA and healthy peers, particularly in forefoot loading, maximum loading, single-limb support duration, and CoM displacement—highlighting the compounded impact of joint inflammation and postural deformities. Taken together, our findings indicate that children with JIA and coexisting scoliosis walk differently compared to healthy peers, but they do not determine whether these deviations are driven predominantly by JIA, scoliosis, or their combined effect.

Our findings showed that children with JIA had significantly lower forefoot loading on both sides, despite similar overall and rearfoot loading values. These results may reflect compensatory gait adaptations to avoid pain or stiffness in the forefoot joints, commonly affected in JIA [18,27]. This proposed mechanism should be interpreted as a working hypothesis rather than a definitive causal pathway. Altered plantar pressure distribution in JIA patients has previously been associated with joint inflammation and limited range of motion, particularly in the ankle and metatarsophalangeal joints, which can shift loading patterns proximally [7]. These results may reflect compensatory adaptations to avoid pain or stiffness in affected joints. This proposed mechanism should be interpreted as a working hypothesis rather than a definitive causal pathway.

The observed decrease in maximum loading and single-limb support (SLS) duration, particularly on the dominant side, supports prior research indicating reduced stability and altered force application during gait in JIA [19,28]. Shortened SLS duration may be a protective strategy to minimize time spent on a painful or unstable limb, contributing to a less efficient gait cycle and increasing energy expenditure.

One of the most notable findings was the increased CoM displacement on the dominant side in the JIA group. Previous studies on JIA and on scoliosis where trunk asymmetry and spinal curvature contribute to instability and exaggerated CoM excursions have separately reported alterations in plantar loading, trunk posture and CoM control, and our results appear broadly consistent with these patterns [27,29,30]; however, the present data cannot be used to directly attribute specific gait features to either condition alone. Given the presence of secondary scoliosis in our JIA cohort, these children may face dual biomechanical burdens: joint-related asymmetries from arthritis and spinal-induced postural deviations. This interaction likely exacerbates gait instability and balance challenges. However, this explanation remains hypothetical and should be tested in future longitudinal and mechanistic studies.

Interestingly, while temporal and loading asymmetries were evident, walking speed did not differ between groups. This suggests that children with JIA may prioritize maintaining overall ambulation pace at the expense of altered gait mechanics—a phenomenon described in other pediatric rheumatology studies [18]. However, maintaining normal speed does not equate to biomechanical efficiency and may further stress joints in the long term.

Our findings also reflect and expand upon the work by Kisa et al. [31], who demonstrated that scoliosis-specific exercise interventions in JIA can improve posture and functional outcomes. The gait deviations noted in our study underline the importance of integrating gait analysis into routine assessment and rehabilitation planning. Exercise programs aimed at improving forefoot function, trunk stability, and weight transfer could potentially reduce CoM displacement and improve temporal symmetry. Our findings are consistent with previous reports showing that scoliosis-specific or JIA-focused exercise programs can improve posture and functional outcomes, but the present study did not evaluate any intervention, and the potential impact of targeted rehabilitation on plantar loading and CoM displacement remains to be tested in controlled trials.

It is also worth noting that disorders mimicking JIA, such as progressive pseudorheumatoid dysplasia (PPRD), can present with similar gait abnormalities due to skeletal dysplasia [32]. This highlights the necessity of comprehensive biomechanical evaluation and differential diagnosis in children presenting with both joint symptoms and gait disturbances.

In the JIA group, joint involvement predominantly affected the lower limbs, particularly the ankle and knee, while hip and upper limb joints were less frequently involved, as summarized in Table 1. Disease-specific variables, including affected joint distribution and scoliosis characteristics, are therefore presented descriptively for the JIA group, whereas between-group statistical comparisons were limited to demographic and gait-related parameters.

A key strength of this study is its novelty; to our knowledge, this is the first investigation to specifically examine gait characteristics in children diagnosed with both JIA and secondary scoliosis. By focusing on this comorbid population, the study addresses a significant gap in the pediatric rheumatology and biomechanics literature. The use of objective gait analysis methods—including plantar pressure distribution and CoM displacement—provides detailed insights into functional impairments that are not easily captured through clinical observation alone.

This study has several limitations. First, its cross-sectional design precludes causal inferences between JIA, scoliosis and the observed gait deviations. Second, although plantar pressure and baropodometric analyses provide objective data, assessments were not blinded, which may have introduced observer bias despite predefined trial selection criteria. Third, walking speed was not strictly standardized; participants walked at their preferred pace, which reflects real-life conditions but may have influenced temporal and loading parameters. Furthermore, detailed information on individual DMARD/corticosteroid regimens and standardized composite disease activity scores were not available for all participants, limiting the exploration of direct associations between pharmacological treatment, disease activity and gait parameters. In addition, although several gait parameters were analyzed, no formal correction for multiple testing was applied, which may increase the risk of type I error and requires cautious interpretation of marginally significant findings. Moreover, because the study did not include groups with JIA without scoliosis or scoliosis without JIA, the specific contribution of each condition to the observed gait alterations cannot be disentangled, and our findings should be interpreted as describing the overall pattern in children with coexisting JIA and scoliosis rather than the isolated effects of either diagnosis. Finally, only children with oligoarticular JIA and mild to moderate scoliosis were included, restricting generalizability to other JIA subtypes and to more severe spinal deformities; larger multi-center studies in more diverse populations are needed to confirm these results.

## 5. Conclusions

Children with JIA and scoliosis exhibit clear deviations in gait, particularly involving forefoot loading, loading symmetry and trunk control, when compared with healthy peers. These findings underscore the value of baropodometric gait analysis for detecting subtle functional alterations in this population and for informing individualized rehabilitation planning. However, due to the cross-sectional design, this study does not allow us to draw conclusions about causality or long-term functional consequences of these gait deviations. Future longitudinal studies are needed to determine how gait characteristics evolve over time and to evaluate whether targeted exercise and rehabilitation programs can modify plantar loading patterns, CoM displacement and functional mobility in children with JIA and secondary scoliosis.

## Figures and Tables

**Table 1:** Demographic and clinic characteristics of participants.

	JIA (n = 25) n (%)	Healthy Controls (n = 25) n (%)	*p*
Sex (n)	25	25	0.54
Female	18 (72)	16 (64)	
Male	7 (28)	9 (36)	
Age (years)	11.76 ± 2.26 (8–16)	11.42 ± 2.67 (8–16)	0.57
BMI (kg/m^2^)	20.18 ± 2.19 (14.90–26.20)	19.12 ± 3.11 (14.20–28.00)	0.53
Disease duration (months)	58.40 ± 22.09 (12–120)	-	
Affected joint (n)			
Ankle	9 (36)	-	
Knee	24 (56)	-	
Hip	-	-	
Hand-wrist	2 (8)	-	
Elbow	-	-	
Distribution of major curvatures			
Torakal	12 (48)	-	
Lomber	11 (44)	-	
Torakolomber	2 (8)	-	
ATR (°)	6.82 ± 1.48 (4–9)	-	
Cobb angle (°)	16.96 ± 3.12 (12–24)	-	

Abbreviations: JIA, juvenile idiopathic arthritis; BMI, body mass index; kg, kilogram; m, meter; ATR, angle of rotation.

**Table 2:** Comparison of the interested parameters between the groups.

Parameters		JIA (n = 25) Mean ± SD Min–Max	Controls (n = 25) Mean ± SD Min–Max	*p*	Effect Size Cohen’s d
Forefoot loading (%)	Dominant	15.95 ± 8.36 (3–38)	19.32 ± 8.77 (5–27)	**0.028**	**0.39**
	Non-Dominant	15.60 ± 8.76 (2–36)	19.88 ± 8.82 (2–33)	**0.026**	**0.48**
Rearfoot loading (%)	Dominant	33.96 ± 10.11 (11–49)	34.16 ± 7.82 (5–40)	0.611	0.02
	Non-Dominant	34.47 ± 9.67 (15–58)	35.44 ± 8.65 (15–47)	0.242	0.10
Overall loading (%)	Dominant	49.92 ± 6.36 (35–62)	51.54 ± 8.18 (39–73)	0.590	0.22
	Non-Dominant	50.02 ± 6.38 (38–65)	50.71 ± 6.21 (26–63)	0.133	0.11
Maximum loading (%)	Dominant	83.26 ± 20.60 (50–110)	85.47 ± 24.56 (65–100)	**0.041**	**0.09**
	Non-Dominant	80.12 ± 18.55 (51–108)	84.27 ± 23.15 (49–109)	**0.042**	**0.19**
SLS duration (msec)	Dominant	355.16 ± 61.26 (290–405)	371.19 ± 60.88 (301–417)	**0.027**	**0.26**
	Non-Dominant	347.60 ± 59.19 (295–401)	354.59 ± 62.20 (290–406)	0.081	0.11
Walking speed (km/h)		4.73 ± 0.88 (3–6)	4.98 ± 1.25 (3–6)	0.862	0.23
CoM Displacement (mm)	Dominant	186.32 ± 24.98 (144–220)	171.31 ± 28.67 (101–230)	**0.044**	**0.55**
	Non-Dominant	174.22 ± 37.47 (63–225)	175.19 ± 35.97 (60–224)	0.841	0.02

Abbreviations: JIA, juvenile idiopathic arthritis; SD, standard deviation; min, minimum; max, maximum; msec, millisecond; km, kilometer; h, hour; SLS, Single-limb stance; CoM, center of mass; mm, millimeter. The bold values indicate statistically significant differences.

## Data Availability

The data that support the findings of this study are available from the corresponding author, [G.L.] due to privacy, upon reasonable request.
